# Prediction of Cardiovascular Risk Using Framingham, ASSIGN and QRISK2: How Well Do They Predict Individual Rather than Population Risk?

**DOI:** 10.1371/journal.pone.0106455

**Published:** 2014-10-01

**Authors:** Tjeerd-Pieter van Staa, Martin Gulliford, Edmond S.-W. Ng, Ben Goldacre, Liam Smeeth

**Affiliations:** 1 Health eResearch Centre, Farr Institute for Health Informatics Research, University of Manchester, Manchester, United Kingdom; 2 Department of non-communicable diseases, London School of Hygiene & Tropical Medicine, London, United Kingdom; 3 Utrecht Institute for Pharmaceutical Sciences, Utrecht University, Utrecht, the Netherlands; 4 Primary Care and Public Health Sciences, King's College, London, United Kingdom; Universidad Peruana de Ciencias Aplicadas (UPC), Peru

## Abstract

**Background:**

The objective of this study was to evaluate the performance of risk scores (Framingham, Assign and QRISK2) in predicting high cardiovascular disease (CVD) risk in individuals rather than populations.

**Methods and findings:**

This study included 1.8 million persons without CVD and prior statin prescribing using the Clinical Practice Research Datalink. This contains electronic medical records of the general population registered with a UK general practice. Individual CVD risks were estimated using competing risk regression models. Individual differences in the 10-year CVD risks as predicted by risk scores and competing risk models were estimated; the population was divided into 20 subgroups based on predicted risk. CVD outcomes occurred in 69,870 persons. In the subgroup with lowest risks, risk predictions by QRISK2 were similar to individual risks predicted using our competing risk model (99.9% of people had differences of less than 2%); in the subgroup with highest risks, risk predictions varied greatly (only 13.3% of people had differences of less than 2%). Larger deviations between QRISK2 and our individual predicted risks occurred with calendar year, different ethnicities, diabetes mellitus and number of records for medical events in the electronic health records in the year before the index date. A QRISK2 estimate of low 10-year CVD risk (<15%) was confirmed by Framingham, ASSIGN and our individual predicted risks in 89.8% while an estimate of high 10-year CVD risk (≥20%) was confirmed in only 48.6% of people. The majority of cases occurred in people who had predicted 10-year CVD risk of less than 20%.

**Conclusions:**

Application of existing CVD risk scores may result in considerable misclassification of high risk status. Current practice to use a constant threshold level for intervention for all patients, together with the use of different scoring methods, may inadvertently create an arbitrary classification of high CVD risk.

## Introduction

Cardiovascular disease (CVD) is a major cause of mortality and morbidity worldwide. It causes impaired quality of life and accounts for a large share of health services utilization [1]. Statins are widely used medications in the prevention of CVD. A recent Cochrane review reported that statins reduce the risk of mortality by 16% and CVD outcomes by 26% in people without a history of CVD [1]. Most guidelines recommend that statins should only be used in primary prevention in people with a high absolute CVD risk [2], [3]. As an example, the National Collaborating Centre for Primary Care and Royal College of General Practitioners and the National Institute of Clinical Excellence (NICE) recommended in 2007 to use statins “…as part of the management strategy for the primary prevention of CVD for adults who have a 20% or greater 10-year CVD risk of developing CVD…” [2].

A large number of risk assessment tools have been developed to support clinicians in determining the long-term risks of CVD [4]. The Framingham, ASSIGN and QRISK2 risk scores are widely used to predict 10-year CVD risk for primary prevention. The Framingham risk score is based on a US cohort recruited several decades ago [5]. The ASSIGN risk score was derived from the Scottish Heart Health Extended Cohort [6] and the QRISK risk score from a large primary care database in England and Wales [7], [8]. These scores were based on risk factors that can easily be measured in the general population. The Framingham, ASSIGN and QRISK2 risk scores have been validated by comparing observed to predicted risks in the overall population [9]. There is no consensus about what risk score to use for CVD risk assessment and guidelines for primary CVD prevention propose to use any risk score [10]. These three risk scores are currently being used in the UK to determine CVD risk.

A recent review of CVD risk prediction models recommended that claims of improved performance of new models over established models should be documented in several studies carried out by independent investigators [9]. There is little evidence about how accurately these risk scores predict high CVD risk in individuals. A risk score could perform well in the overall population if it consistently predicts low rather than high risks as those at high risks are typically only a minority [11]. The objective of this study was to evaluate the validity and reliability of the Framingham, ASSIGN and QRISK2 scores in predicting individual CVD risk.

## Material and Methods

### Data source

This study used data from the General Practice Research Database in the United Kingdom which is part of the Clinical Practice Research Datalink (CPRD), previously known as the General Practice Research Database. CPRD comprises the computerised medical records maintained by general practitioners (GPs). Almost all people in the UK are registered with a general practice. GPs play a key role in the UK health care system, as they are responsible for primary health care and specialist referrals. The GPs are typically informed by hospitals of diagnoses made during outpatient consultations and hospitalisations. The data recorded in the CPRD since 1987 include demographic information, prescription details, clinical events, preventive care provided, specialist referrals, hospital admissions and their major outcomes [12]. A recent review of validation studies found that medical data in the CPRD were generally of high quality [13]. Fifty-five studies of the CPRD recording of diseases of the circulatory system reported a median percentage of cases confirmed of 85.3% [13]. People in CPRD have now been linked individually and anonymously to the national registry of hospital admission (Hospital Episode Statistics [HES]) and death certificates. The linkages are performed using the patient's unique NHS number, date of birth, sex and postcode of residence. HES collect the dates of hospital admission and discharge and main diagnoses, as extracted from the medical records by coding staff in England. The death certificates list the date and causes of death. Linked data were available for 50% of the CPRD population as, at the time of the study, this only included practices in England willing to provide unique patient identifiers to the Trusted Third Party. The protocol of this study was approved by the CPRD Independent Scientific Advisory Committee.

### Study populations

The main study population consisted of people aged 35–74 years, using the November 2011 version of CPRD and drawn from CPRD practices that participated in the linkages. The start of follow-up was one year after start of the patient's CPRD data collection or 1 January 1998, whichever date came last. HES and death certificates data were available from 1998 onwards. The end of follow-up was the patient's end of CPRD data collection or death. The index date at which the CVD risk assessment was conducted, was a randomly selected date during this period of follow-up. This approach was different from that used in the QRISK2 analysis, which set the index date to 1-1-1998 unless the patient's data collection started later (e.g. due to patient newly registering). The use of a random index date was preferred in order to investigate changes in data recording (a newly registered patient may have different levels of e.g. missing data). The following persons were excluded: (i) those with CVD prior to the index date or with missing dates, (ii) those prescribed a statin prior to the index date or with missing dates, (iii) those temporarily registered with the practice. Follow-up was censored at the date of a first statin prescription.

### Outcomes

The following incident CVD outcomes were included:

- CVD as recorded by the GPs (myocardial infarction, angina, coronary heart disease, stroke and transient ischemic attack).
- hospitalisation due to CVD as recorded by the hospital in HES (either primary or secondary admission diagnostic ICD10 codes): angina pectoris (I20); acute myocardial infarction (I21); complications following acute myocardial infarction (I23); other acute ischaemic heart disease (I24); chronic ischaemic heart disease (I25); cerebral infarction (I63); and stroke, not specified as haemorrhage or infarction (I64), as used for QRISK2 [8]. Additional codes included intracerebral haemorrhage (I61) and other nontraumatic intracranial haemorrhage (I62).
- Death due to CVD as reported on a death certificate (primary or secondary cause). The ICD-10 codes were similar to those used for hospitalisations.

Death due to causes other than CVD was also measured.

### Imputation for missing variables

Missing values for smoking status, systolic blood pressure, ratio of total serum cholesterol and high density lipoprotein (HDL) cholesterol and BMI were imputed (using MI and MIANALYZE imputation procedures in SAS). The imputation regression models included the risk factors as listed in supplementary Table 1, CVD occurrence, death due to causes other than CVD, duration of follow-up and interactions between CVD occurrence and death and duration of follow-up. Five imputation datasets were created and the effect estimates were based on the combination of point and variance estimates from these five datasets [14]. The same imputed values for each patient were used across the different risk scores.

**Table 1:** Baseline characteristics of the study population.

Risk factor		Men (N = 924233)	Women (N = 927953)
Age at index date	Mean (sd)	49.5 (11.3)	50.5 (11.8)
Duration of follow-up after index date	Mean (sd)	3.2 (3.0)	3.4 (3.2)
	≥5 years	221588 (24.0%)	248012 (26.7%)
	≥10 years	37741 (4.1%)	47159 (5.1%)
Ethnicity	White	444617 (48.1%)	538979 (58.1%)
	Black	11699 (1.3%)	14820 (1.6%)
	Indian	8627 (0.9%)	9805 (1.1%)
	Other	17231 (1.9%)	22210 (2.4%)
	Unknown	442059 (47.8%)	342139 (36.9%)
Index of deprivation quintiles	1 (most deprived)	230269 (24.9%)	237408 (25.6%)
	2	221536 (24.0%)	227396 (24.5%)
	3	180771 (19.6%)	182010 (19.6%)
	4	170552 (18.5%)	167548 (18.1%)
	5 (least deprived)	121105 (13.1%)	113591 (12.2%)
Body mass index	Low (<20)	12661 (1.4%)	40105 (4.3%)
	Normal (≥20 - <26)	181156 (19.6%)	273038 (29.4%)
	Overweight (≥26)	249981 (27.0%)	273694 (29.5%)
	Unknown	480435 (52.0%)	341116 (36.8%)
Smoking status	No	264612 (28.6%)	388477 (41.9%)
	Past	120610 (13.0%)	115259 (12.4%)
	Current	193165 (20.9%)	171328 (18.5%)
	Unknown	345846 (37.4%)	252889 (27.3%)
Systolic blood pressure	Recorded	596319 (64.5%)	771566 (83.1%)
	Mean (sd)	133.1 (16.3)	128 (18.0)
Cholesterol HDL ratio	Recorded	158927 (17.2%)	168611 (18.2%)
	Mean (sd)	4.5 (1.4)	3.8 (1.2)
Number of records for medical events in the electronic health records in the year before	Mean (sd)	10 (14.5)	16.5 (17.6)
Treated hypertension		72459 (7.8%)	91125 (9.8%)
Diabetes mellitus		20144 (2.2%)	15778 (1.7%)
Atrial fibrillation		7247 (0.8%)	4351 (0.5%)
Chronic renal disease		4830 (0.5%)	6998 (0.8%)
Rheumatoid arthritis		4280 (0.5%)	10721 (1.2%)
Left ventricular hypertrophy		2297 (0.2%)	1361 (0.1%)

### CVD risk scores

Three risk scores were analysed including Framingham, ASSIGN and QRISK2. We did not analyse the Joint British Society 2 risk score [3] given the similarity to the Framingham risk score. The 10-year CVD risks at the index date as predicted by Framingham and ASSIGN were estimated using the publicly available risk equations [7], [8]. The risks predicted by QRISK2 were calculated using the commercial software program as provided by CLINRISK Limited on a fee-paying licence using the 2012 version [http://qrisk.org/index.php]. The CVD risks as predicted by the risk scores were based on the risk factors measured at the index date. A previous study reported that lifestyle variables as recorded in CPRD (such as obesity and smoking) were important predictors for myocardial infarction [15].

### CVD risks based on a competing risk regression model

We also estimated for each patient the individual long-term CVD risks as modelled by a competing risk Cox proportional hazards regression model [16]. This was done to estimate as accurately as possible the actual CVD risks for each patient in the study population, which could then be compared to the risks as predicted by the risk scores. Competing risk regression was used as standard Cox regression model has been reported to overestimate 10-year CVD risk of coronary heart disease [17]. Accounting for the risks of competing events (such as death due to non-CVD causes) may be important in the frail and older populations as CVD occurrence may be precluded by the development of other diseases. Fractional polynomials were used to model non-linear risk relations with the continuous variables [18]. The regression models were conducted separately by gender and three age groups.

The validation of risk scores involves the measurement of calibration and discrimination. Calibration is the comparison of observed and predicted event rates and discrimination the ability of the risk score to distinguish between people who do and do not experience the event of interest [19]. We assessed calibration by comparing observed (using competing risk life tables) and predicted event rates in subgroups as defined by the vigintiles of predicted risk (vigintiles are the values that divides the distribution of individuals into twenty groups of equal frequency). Discrimination is the extend a risk score is able to differentiate between those who develop the outcome and those who do not. Discrimination is typically assessed by estimating the c index [19]. Rather than estimating this c index which is a global measure and population average, we evaluated the predicted risks at the index date for those people who developed CVD during follow-up. Good discrimination would have occurred if CVD cases mostly developed in those with high predicted risks. External validation is typically recommended for models that need to be generalised to other populations [19]. Our competing risk regression model was not intended to be generalised but only to estimate as best as possible the individual risks in our study population. We also assessed reclassification by evaluating the consistency in prediction between the different risk scores.

### Descriptive analyses

The main analysis consisted of a comparison of the predictions of CVD risk at the index date with the four risk scores for each individual patient. The intraclass correlation coefficients (ICCs) in individual risk prediction between the four risk scores were estimated [20]. We report the ICCs rather than Pearson correlation coefficients because the former provides a measure of agreement between scores while the latter shows how well one score predicts the other. This distinction is important when a threshold (such as 20%) is recommended for deciding the course of clinical intervention.

Two different analyses were conducted in order to evaluate bias with the risk scores. The first analysis concerned secular trend in CVD incidence. CVD incidence has decreased over several decades [21]. Thus, the risk scores may overestimate CVD risks in current practice. In order to estimate the potential effects of this secular trend, incidence rates were measured in each calendar year. The second bias analysis concerned multiple imputations as used in the QRISK2 estimation. This method assumes that the occurrence of missing data is random conditional on other observed patient characteristics. In UK general practice, risk factors are typically not recorded unless the patient visits the practice. People with certain conditions may also be more likely to be screened for risk factors which incur extra payments (Quality Outcome Framework). In order to evaluate the effects of imputation, Cox regression was used to compare the CVD incidence in people with imputed values (for BMI, systolic blood pressure, cholesterol and smoking status) and those with measured values. If the assumption behind multiple imputations is correct, it can be expected that the CVD rate is similar between those with recorded and imputed values (conditional on the other risk factors in the model). SAS version 9.2 was used for the analyses.

## Results

The study population included 1.8 million persons with an average follow-up of 3.3 years (Table 1). Ethnicity was not recorded for about half of the men and one-third of the women. About one-quarter of the study population had a follow-up after the random date of at least 5 years. Women were more likely to have information on smoking status, BMI and systolic blood pressure. The extent of missing data decreased sharply over calendar time. In 1998, BMI was missing in 47.3% of people, smoking status in 47.3%, systolic blood pressure in 33.4% and cholesterol/HDL ratio in 97.8%; in 2010, these figures were 32.7%, 14.3%, 22.3% and 72.2%, respectively.

CVD outcomes occurred in 69,870 persons. Major risk factors for CVD included number of cigarettes smoked per day of 21+ (relative rate [RR] = 2.77 [95% CI 2.54–3.03] in women and RR = 2.45 [95% CI 2.32–2.59] in men), unknown ethnicity (RR = 0.46 [95% CI 0.45–0.48] and RR = 0.42 [95% CI 0.42–0.43]) and 50+ records in CPRD in the year before (RR = 5.75 [95% CI 5.33–6.20] and RR = 4.31 [95% CI 4.02–4.62]).

The CVD incidence decreased over calendar time. The age- and sex-adjusted RR of CVD was 0.61 (95% CI 0.59–0.63) in 2010 compared to 1998. This RR was 0.94 (95% CI 0.89–1.00) for hospitalisations due to CVD (as recorded in HES) and 0.52 (95% CI 0.49–0.54) for GP-recorded CVD. Death due to CVD (as recorded on death certificates) also decreased over calendar time (RR of 0.58 [95% CI 0.52–0.64]).

The calibration of the competing risk model showed small differences on average with observed risks across vigintiles of risk. The largest difference between predicted and observed CVD risk occurred in the vigintile with highest risk (predicted 10-year risk of 35.9% compared to an observed risk of 34.9%). The differences between observed and predicted 10-year risks were on average less than 0.2% in 16 vigintiles with lowest risk.

Table 2 shows the distribution of 10-year CVD risks as estimated by competing risk regression. In people aged 50 years or older, 22.9% had a 10-year CVD risk of ≥20% and 51.5% of risk of ≥10%. The risks varied considerably in this age group: the 5^th^ percentile of 10-year CVD risk was 1.4% and 95^th^ percentile 34.6%.

**Table 2:** Distribution of 10-year CVD risk as estimated for individual persons in the study population using competing risk regression stratified by age and gender.

		Number of persons (%) in categories of 10-year CVD risk				10-year CVD risk			
Risk factor		Risk of <10%	Risk of 10-15%	Risk of 15–20%	Risk of ≥20%	Mean	Median	5^th^ percentile	95^th^ percentile
All		1373431 (74.2%)	165004 (8.9%)	111837 (6.0%)	201914 (10.9%)	7.4	3.3	0.2	27.9
Age at index	35–49	959377 (96.0%)	24718 (2.5%)	8981 (0.9%)	6196 (0.6%)	2.4	1.2	0.2	8.9
	50+	414054 (48.5%)	140286 (16.4%)	102857 (12.1%)	195718 (22.9%)	13.2	10.4	1.4	34.6
Gender	Men	629258 (68.1%)	91544 (9.9%)	65487 (7.1%)	137944 (14.9%)	9.1	4.7	0.3	31.7
	Women	744173 (80.2%)	73460 (7.9%)	46351 (5.0%)	63970 (6.9%)	5.7	2.2	0.2	22.8

The level of agreement in CVD risk prediction was best between ASSIGN and QRISK (intraclass correlation coefficient of 0.93) and lowest between Framingham and estimated risks in CPRD (0.77). The correlation was 0.91 between Framingham and ASSIGN, 0.87 between Framingham and QRISK2, 0.80 between ASSIGN and estimated risks in CPRD and 0.84 between QRISK2 and estimated risks in CPRD. As shown in Table 3, the difference in the predicted 10-year CVD risks between QRISK2 and the risks predicted based a competing risk model was on average 0.4%, while the predicted 10-year CVD risk with Framingham was on average 2.3% higher and ASSIGN 1.4% higher compared to that predicted by the competing risk model. When analysing the concordance in estimates for individual persons, only 55.6% of persons had a small difference in the risks predicted by QRISK2 and the risks based on the competing risk model.

**Table 3:** Concordance between the risks predicted by the different risk scores and those estimated by competing risk regression in CPRD.

					Difference in individual risk predictions						
					≤ −10%	Between −5 and −10%	Between −5 and −2%	Between −2 and +2%	Between +2 and +5%	Between +5 and +10%	≥ +10%
Comparator risk method	Reference risk method	Mean predicted risk by comparator risk method	Mean predicted risk by reference risk method	Mean difference between the two methods	% of persons	% of persons	% of persons	% of persons	% of persons	% of persons	% of persons
Framingham risk score	ASSIGN risk score	9.7	8.8	0.9	0.8	3.4	4.4	65.4	16.5	7.3	2.2
	QRISK2 risk score	9.7	7.8	1.9	1.4	3.7	5.2	46.3	25.8	14.1	3.5
	Individual risks estimated in CPRD using competing risk regression	9.7	7.4	2.3	2.9	4.9	6.2	39.1	22.8	16	8.1
ASSIGN risk score	Framingham risk score	8.8	9.7	−0.9	2.2	7.3	16.5	65.4	4.4	3.4	0.8
	QRISK2 risk score	8.8	7.8	1	1	2.5	6	61.5	24.1	3.7	1
	Individual risks estimated in CPRD using competing risk regression	8.8	7.4	1.4	3.6	5.5	6.8	41.7	27	11.3	4.2
QRISK2 risk score	Framingham risk score	7.8	9.7	−1.9	3.5	14.1	25.8	46.3	5.2	3.7	1.4
	ASSIGN risk score	7.8	8.8	−1	1	3.7	24.1	61.5	6	2.5	1
	Individual risks estimated in CPRD using competing risk regression	7.8	7.4	0.4	3.7	6.3	8.8	55.6	14.3	7.9	3.5

Table 4 shows the differences between Framingham, ASSIGN and QRISK2 compared to the estimated risks in CPRD stratified by the risk factors. The mean differences between QRISK2 predicted risks and estimated risks in CPRD increased by age. QRISK2 overestimated 10-year CVD risk by 2.2% in people aged ≥65 years compared to the risks estimated in CPRD while QRISK2 predicted and CPRD estimated risks were, on average, similar in younger people. The concordance between QRISK2 predicted risks and the estimated risks in CPRD changed over calendar time; QRISK2 underestimated 10-year CVD risk by 3.2% in 1998–2001 and overestimated risk by 2.2% in 2006–2010 compared to the estimated risks in CPRD. Larger deviations between the risks predicted by QRISK2 and risks estimated in CPRD occurred with different ethnicities, diabetes mellitus, left ventricular hypertrophy and number of records for medical events in the electronic health records in the year before the index date.

**Table 4:** Differences in individual risk predictions between risk scores and risks estimated using competing risk regression stratified by risk factors (selected results for larger differences).

		Framingham risk score		ASSIGN risk score		QRISK2 risk score	
Risk factor		Mean difference with risks estimated using competing risk regression	Percentage of persons with difference between −2 and +2%	Mean difference with risks estimated using competing risk regression	Percentage of persons with difference between −2 and +2%	Mean difference with risks estimated using competing risk regression	Percentage of persons with difference between −2 and +2%
Gender	Men	3.7	24.0	1.7	24.8	0.4	47.5
	Women	0.8	54.2	1.0	58.5	0.4	63.6
Age at index	35–44	2.0	58.9	1.5	59.9	0	84.4
	45–54	3.2	31.8	1.2	39.6	0	53.3
	55–64	2.8	22.2	0.5	24.1	0.3	26.5
	65–74	0.7	19.6	2.4	18.2	2.2	18.5
Calendar year index	1998–2000	−0.7	32.0	−1.7	34.6	−3.2	42.3
	2001–2003	1.6	38.0	0.6	39.8	−0.5	49.8
	2004–2006	2.8	41.2	1.9	44.3	1.0	57.5
	2007–2009	3.6	41.6	2.8	44.7	2.1	62.4
	2010–2011	3.8	42.3	3.1	44.0	2.2	66.1
Ethnicity	White	0	46.9	−0.7	51.1	−1.5	53.3
	Black	2.6	52.0	2.0	56.1	0	77.6
	Indian	2.5	46.2	−0.7	62.7	−0.2	64.7
	Unknown	5.1	27.9	4.1	27.9	2.8	56.8
Diabetes mellitus	no	2.2	39.6	1.2	42.2	0.3	56.3
	yes	3.3	17.6	7.7	13.3	3.6	18.7
Left ventricular hypertrophy	no	2.3	39.2	1.4	41.7	0.4	55.7
	yes	1.1	12.1	−15.2	11.4	−11.9	13.3
Number of records for medical events in the electronic health records in the year before	0	4.9	26.7	3.4	26.3	1.6	58.0
	1–3	3.9	34.8	2.7	36.3	1.1	60.2
	4–8	2.8	42.3	1.8	45.6	0.5	60.8
	9–18	1.7	43.6	0.9	47.4	0.1	55.3
	19–4	0.1	43.5	−0.3	46.9	−0.5	48.7
	50+	−2.6	37.3	−2.7	38.7	−1.9	37.6

**Figure 1:**
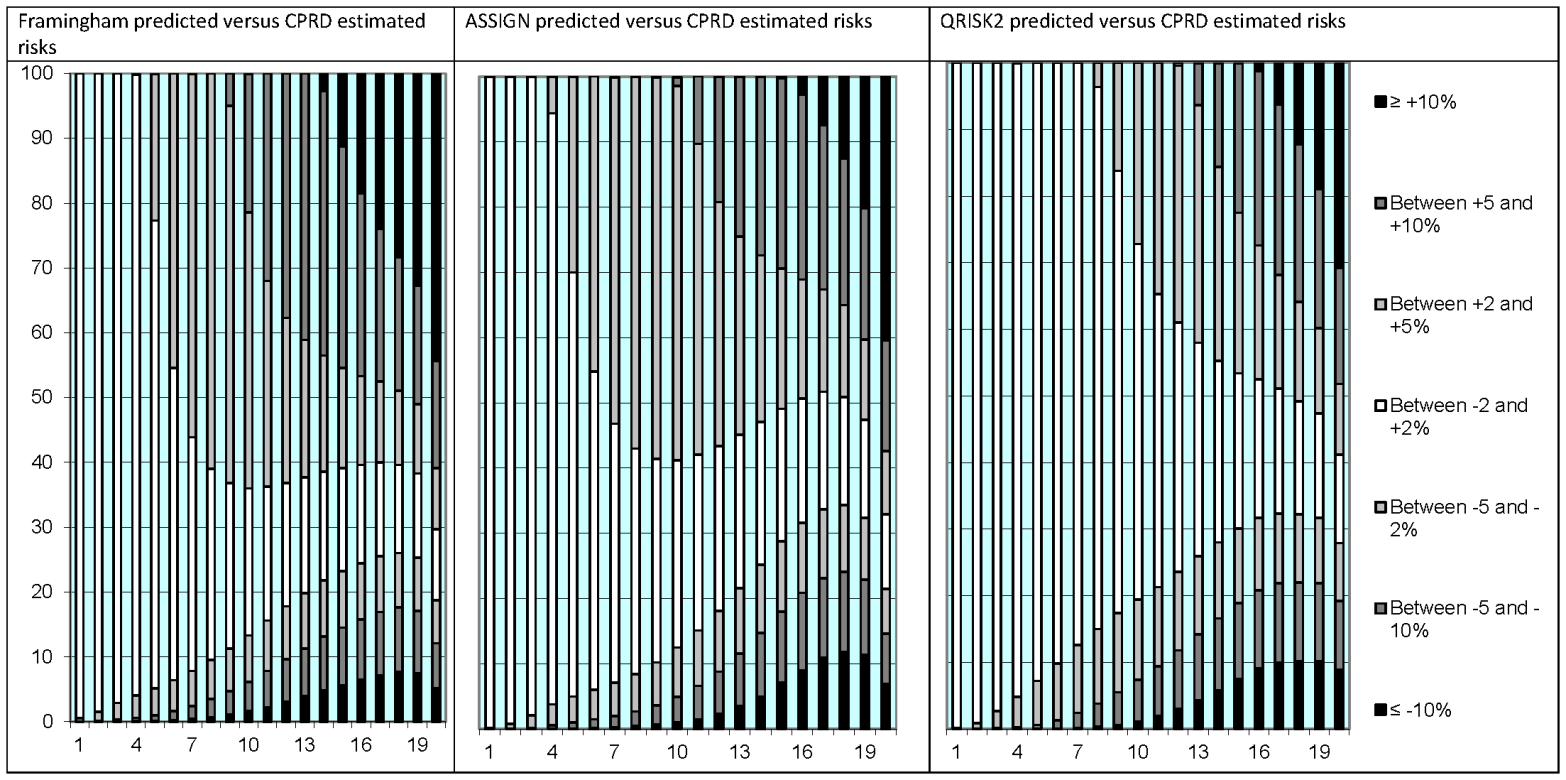
Absolute differences in individual 10-year CVD risk prediction between the Framingham, ASSIGN and QRISK2 risk scores and the individual risks estimated in CPRD using competing risk regression stratified by vigintiles of predicted risk. X-axis: Vigintiles of predicted risk. Y-axis: Percentage of persons.

The differences in individual risk prediction between the risk scores were largest among people with higher CVD risks (Figure 1). In the lowest vigintile of risk, the risk predictions by QRISK2 were similar to the individual risks estimated in CPRD (absolute difference of less than 2%) for 99.9% of people; in the highest vigintile of risk, this was only 13.3%.

The risk scores predicted low 10-year CVD risk fairly consistently (Table 5). A QRISK2 estimate of low 10-year CVD risk (<15%) was confirmed by Framingham, ASSIGN and the CPRD estimated risks in 89.8% of people. An estimate of high 10-year CVD risk (≥20%) by QRISK2 was confirmed in 48.6% of people.

**Table 5:** Consistency in risk predictions with risk scores or competing risk regression in predicting high and low CVD risk.

				Number of other risk scores with 10-year CVD risk of ≥20%	
		0	1	2	3
Reference risk method	≥20%	% of persons	% of persons	% of persons	% of persons
Framingham risk score		18.2	15.7	24.2	41.9
ASSIGN risk score		4.6	14.2	30.1	51.1
QRISK2 risk score		7.6	15.1	28.6	48.6
Individual risks estimated using competing risk regression		21.6	15.2	14.5	48.7
				Number of other risk scores with 10-year CVD risk of <15%	
		0	1	2	3
	<15%	% of persons	% of persons	% of persons	% of persons
Framingham risk score		0.6	2.3	3.5	93.5
ASSIGN risk score		0.4	2.2	8.2	89.2
QRISK2 risk score		0.4	2.6	7.3	89.8
Individual risks estimated using competing risk regression		3.8	3.1	5.9	87.2

The majority of CVD cases occurred in people who had a predicted 10-year CVD risk of less than 20% (Table 6). Only 41.1% of the cases were predicted by QRISK2 to have a 10-year CVD risk of ≥20% and 27.5% of the cases a 10-year CVD risk of less <10%.

**Table 6:** 10-year CVD risk at index date for persons who developed CVD after the index date (cases).

	10-year CVD risk at the index date#			
	<10%	10–15%	15–20%	≥20%
Risk method	N cases (%)	N cases (%)	N cases (%)	N cases (%)
Framingham risk score	15336 (21.9%)	13087 (18.7%)	11544 (16.5%)	29903 (42.8%)
ASSIGN risk score	18100 (25.9%)	12633 (18.1%)	11516 (16.5%)	27621 (39.5%)
QRISK2 risk score	19212 (27.5%)	10604 (15.2%)	11348 (16.2%)	28705 (41.1%)
Individual risks estimated using competing risk regression	14361 (20.6%)	9662 (13.8%)	10517 (15.1%)	35330 (50.6%)

# Rounded averages across the five imputation datasets.

## Discussion

We found that all three risk scores (Framingham, ASSIGN and QRISK2) predicted the presence of low CVD risk consistently in individual persons. However, predictions of high CVD risk for individuals varied substantively between the risk scores and treatment strategies could be different depending on which risk score is being used. Most CVD cases occurred in people deemed to be at low CVD risk.

Population averages can hide substantial variability in prediction among individual persons and poor prediction of ‘high risk’ status as these estimates are often determined by the large majority of low risk individuals. As succinctly stated by Rose, the ability to estimate the average risk for a group may not be matched by any corresponding ability to predict which individuals are going to fall ill soon [11]. The present study confirmed Rose's observations for CVD prediction, with a considerable variability between risk scores in the prediction of high CVD risk and with most CVD cases occurring in people classified to have lower CVD risk.

The QRISK2 score was developed in a similar setting as the present study and the statistical methods were also broadly similar. As expected, we found that the averages of QRISK2 estimates and our competing risk predictions were reasonably consistent. Two validation studies of QRISK2 reported that the predicted and observed risks were on average similar and they concluded that QRISK2 was accurate in identifying a high risk population [22], [23]. Our analyses of averages support these studies. But we also conducted analyses of individual risk predictions and reached opposite conclusions. We found substantial deviations between the QRISK2 estimates and our competing risk predictions. This was related to the inclusion into the competing risk models of several risk factors, as pre-defined in the protocol, which were found to be strong predictors of risk (such as calendar year, number of GP visits, region and indicators of missing data including ethnicity). Our approach, although not commonly used, allows for an examination of the performance of risk scores in individual persons rather than testing averages across populations. This regression approach should provide, as long as the model is specified correctly, a close representation of the observed risks across multiple risk factors. The regression models also included risk factors not used by the published risk scores, such as number of GP visits (e.g. there was a five-fold difference in CVD risk between women with frequent and no GP attendance). If the risk scores are to be used for individual risk prediction, the evaluation of performance should go beyond population averages.

A recent meta-analysis reported that statins reduced CVD risk mortality in people with low CVD risk. It concluded that the threshold for statin treatment should be reduced to a 10-year CVD risk of 10% [24]. A commentary of this study proposed that statins should be used by all by the age of 50 years as most people aged 50 years or older have higher risks. It stated, incorrectly, that 83% of the men older than 50 years and 56% of women older than 60 years have a 10-year CVD risk of ≥10% [25]. It is questionable whether whole populations should be treated if individual risks vary greatly, as with CVD. Another question is how to deal with individuals who were not eligible for the trials (e.g. a 50-year old with normal LDL and C-reactive protein). There is no guarantee that the treatment effects as observed in trials can be generalised to populations different from those in the trials [26].

The strength of this study was the large size and representativeness of the study population, the well-documented data quality of CPRD [13] and the availability of linked hospital and death certificate data. There are several important limitations. Information on laboratory and physical measurements was missing for a large number of people. The extent of missing data decreased substantially over time. Reasons for this decrease include the availability of electronic communication between practices and laboratories and the incentivisation of practices in measuring and recording of data. We applied imputation techniques but found that people with imputed values had different CVD risks compared to those without missing data. This is not unexpected as healthy people are less likely to visit their practice. Another limitation of this study concerned the use of socioeconomic status in the evaluation of the ASSIGN score. This score used the Scottish IMD and their values cannot be generalised to other regions in the UK. Our approach of standardising English to Scottish IMD may have introduced bias but the direction of bias is likely to have underestimated differences as socioeconomic status is, on average, higher in England. The recording of ethnicity in CPRD also has limitations as there was a substantive discrepancy in ethnicity between CPRD and HES in the recording of ethnicity. Another limitation is that the coding by practices of CVD has changed over calendar time [27] which may explain part of the trend of lower CVD rates over time. However, a secular trend was also observed with hospitalisations recorded in HES and death certificates. The main analyses in this study concerned comparisons of predictions by the different risk scores which are not affected by changes in CVD recording. Another consideration in this study was the use of a random index date rather than one based on the start of data collection. This approach reduced statistical power. However, our rationale for this was the objective to emulate the performance of risk scores in actual clinical practice, with assessments being done at arbitrary dates rather than at the start of data collection. There have also been major changes in the completeness of data recording over time: an imputation model that used an index date of 1-1-1998 did not converge due to high levels of missing cholesterol levels. The number of people with a follow-up exceeding 10 years was also larger than that in the studies for ASSIGN, Framingham and QRISK1 [5], [6], [8].

In conclusion, the Framingham, ASSIGN and QRISK2 risk scores do not predict the presence of high CVD risk well and consistently. Current practice to use any risk score in conjunction with a constant threshold level has inadvertently created an arbitrary classification of high CVD risk. Risk prediction strategies should be based on statistical models that are transparent, derived from a similar population, with data collected recently and updated regularly.
